# Down-regulated *HSDL2* expression suppresses cell proliferation and promotes apoptosis in papillary thyroid carcinoma

**DOI:** 10.1042/BSR20190425

**Published:** 2019-06-04

**Authors:** Jing Zeng, Xiao Ma, Jinjing Wang, Ran Liu, Yun Shao, Yanwei Hou, Zhiyuan Li, Yi Fang

**Affiliations:** 1 Department of Endocrinology, The Fifth Medical Center, Chinese PLA General Hospital (Former 307th Hospital of the PLA), Beijing 100071, China; 2 Key Laboratory of Carcinogenesis and Translational Research, Department of Head and Neck, Perking University Cancer Hospital and Institute, Beijing 100142, China; 3 Department of Pathology, The Fifth Medical Center, Chinese PLA General Hospital (Former 307th Hospital of the PLA), Beijing 100071, China

**Keywords:** apoptosis, HSDL2, NFATc2, Proliferation, protein kinase B, PPP3CA

## Abstract

Papillary thyroid carcinoma (PTC) is the most common type of thyroid cancer. Hydroxysteroid dehydrogenase like 2 (*HSDL2*) can regulate lipid metabolism and take part in cell proliferation. The purpose of the present study was to explore functional role of *HSDL2* gene in PTC. The expression of HSDL2 protein in PTC tissues was estimated using immunohistochemistry analysis (IHC). *HSDL2* mRNA level was detected through quantitative real-time polymerase chain reaction (qRT-PCR). Effects of *HSDL2* gene on cell proliferation and apoptosis were assessed using the shRNA method for both *in vitro* and *in vivo* experiments. Potential target genes of *HSDL2* were determined via bioinformatics analyses and Western blotting. *HSDL2* was up-regulated in PTC tissues and cell lines compared with the controls (all *P*<0.05). Inhibiting HSDL expression could suppress PTC cell proliferation and cycle, and promote apoptosis *in vitro. In vivo*, the knockdown of *HSDL2* gene could significantly suppress tumor growth (all *P*<0.05). Furthermore, *AKT3, NFATc2* and *PPP3CA* genes might be potential targets of *HSDL2* in PTC. *HSDL2* expression was increased in PTC tissues and cells, which could promote tumor progression *in vitro* and *in vivo*.

## Introduction

Papillary thyroid carcinoma (PTC) is the most common subtype of thyroid cancer. Its incidence exhibits continual increases in recent decades worldwide [1]. PTC is more frequently observed in individuals of 20–55 years, especially among females [2]. Early diagnosis remains a great challenge in PTC, due to its asymptomatic nature at early stages. Fine needle aspiration biopsy (FNA) and ultrasound imaging are two commonly used methods for PTC diagnosis, which have relatively high accuracy [3,4]. Overall survival of PTC cases is significantly improved due to great advancements in therapeutic strategies, such as surgery, chemotherapy and radiotherapy [5]. However, the recurrence rate of PTC remains high, gravely weakening the effects of PTC treatment. This condition could partially be explained by increases in incurable cases and by limited understanding on the malignancy-related biological behaviors [6,7]. Therefore, exploring molecular mechanisms of PTC progression is critical to determine therapeutic targets for this malignant disease.

Short-chain dehydrogenases/reductases (SDRs) superfamily contains a series of crucial oxidoreductases [8]. The family plays important roles in catalyzing the oxidation and reduction of various substrates, such as steroids, sugars, retinoids and fatty acids. Previous data have indicated that SDR enzyme dysfunction may influence metabolism, neural development, transformation, thus leading to diseases, such as obesity-related medical conditions, Alzheimer’s disease and cancers [9–11]. As a member of SDRs family, hydroxysteroid dehydrogenase like 2 (*HSDL2*) consists of a C-terminal SCP2-like domain and an N-terminal SDR domain [12]. *HSDL2* has been identified as an effective fatty acid regulatory factor in lipid metabolism [13]. Lipid metabolism is an established hallmark in various human cancers [14–16]. For examples, the study by Hosokawa et al. [17] demonstrated that altered lipid metabolism was correlated with malignant transformation. The level of phosphatidylcholine (32:1) could be employed as a biomarker for the recurrence of triple-negative breast cancer [17]. Lipids could provide energy for membrane formation and realize other functions for aggressively proliferating tumor cells [18]. Additionally, lipid metabolism may play important roles in the activation of essential cell-signaling pathways in carcinogenesis, thus contributing to primary tumor initiation and distant metastasis [19]. As an important regulator for lipid metabolism, *HSDL2* has also been proved to participant in tumorigenesis. It was reported that *HSDL2* could mediate cell proliferation and tumor growth in glioma via Akt-associated signaling pathway. The expression pattern of *HSDL2* was positively correlated with aggressive progression of glioma [14]. However, the study carried out by Zhang et al. [20] reported that overexpression of *HSDL2* resulted in tumor suppressive effects on progression of cholangiocarcinoma via inhibiting cell growth and promoting cell apoptosis. *HSDL2* might play diverse roles in different types of cancer. However, the effects of *HSDL2* on PTC were rarely reported in the past.

In the present study, we aimed to investigate the expression patterns of *HSDL2* gene in PTC tissues and cell lines, as well as its functional roles in PTC progression.

## Methods and materials

### Patients and tissue sample collection

PTC tissues and adjacent normal ones were collected from 17 patients, who were pathologically diagnosed with PTC at Affiliated Hospital of the Academy of Military Medical Sciences. None of the patients had received any anti-tumor therapies prior to the sampling. After the collection, the tissues were immediately stored in liquid nitrogen, and then kept at −80°C for further use. Experimental procedures were accomplished in accordance with the guidelines released by the Ethics Committee of Affiliated Hospital of the Academy of Military Medical Sciences. Signed written informed consent was obtained from each patient.

### Cell culture and transfection

The cells K1 and Nthy-ori 3-1 were purchased from European Collection of Authenticated Cell Cultures (ECACC), while B-CPAP was purchased from the Stem Cell Bank, Chinese Academy of Sciences. Two PTC cell lines (K1: ECACC 92030501 and B-CPAP: SCSP (stem cell storage platform) 543) and human thyroid follicular epithelial cell line (Nthy-ori 3-1: ECACC 90011609) were used for subsequent cell experiments. These cell lines were cultured in RPMI-1640 medium containing 10% fetal bovine serum (FBS) (Gibco, Gaithersburg, U.S.A.). Cell cultures were incubated in a humidified chamber with 5% CO_2_ at 37°C. Cell morphology was performed for further identification of these cells.

Lentiviral vector GV115 carrying shRNA targeting *HSDL2* (sh*HSDL2*) was transferred into K1 and B-CPAP cell lines, so as to down-regulate *HSDL2* expression. Corresponding empty GV115 vector (shCtrl) was used as negative control. Transfection was performed via Lipofectamine 2000 (Life Technologies, Carlsbad, CA, U.S.A.) following the instructions of the manufacturer. Transfection efficiency was estimated employing relative expression of *HSDL2* mRNA in the transfected cells which was detected using quantitative real-time polymerase chain reaction (qRT-PCR).

### Immunohistochemistry analysis

Expression levels of *HSDL2* protein in PTC and adjacent normal tissues were evaluated using immunohistochemistry analysis (IHC). The tissues were fixed by formaldehyde and embedded by paraffin. Then paraffin sections were deparaffinized in xylene and rehydrated in graded alcohols. In order to quench the activity of endogenous peroxidase, the sections were treated using 3% hydrogen peroxide. Later, the activities of antigens were recovered adopting citrate buffer (pH = 6.1) at 95°C for 15 min. The sections were blocked with normal goat serum at 37°C for 10 min, and then incubated with a polyclonal goat anti-*HSDL2* antibody (diluted 1:200, Santa Cruz Biotechnology, CA, U.S.A.) overnight at 4°C. After rinsing with phosphate buffer solution (PBS), the sections were incubated with the second antibody (rabbit anti-goat antibody) at 37°C for 30 min. Last, the sections were incubated in the streptavidin–horseradish peroxidase complex. Staining results were reviewed and scored by two independent observers. Staining intensity was scaled as 0 (no staining), 1 (weak staining), 2 (moderate staining) and 3 (strong staining). The proportion of positively stained tumor cells was scored as 0 (0%), 1 (<25%), 2 (26–50%), 3 (51–75%) and 4 (>75%). Final score was calculated using staining intensity score and the proportion of positive tumor cells. Three fields were randomly selected under microscope for each specimen, and their average values were used for final analysis.

### RNA extraction and qRT-PCR

Total RNA was extracted from PTC cell lines (K1 and B-CPAP) and from human normal thyroid cell line (Nthy-ori 3-1) using TRIzol reagent (Invitrogen, Carlsbad, CA, U.S.A.) according to the manufacturer’s instructions. RNA quality and concentration were determined using Nanodrop 2000 (Wilmington, DE 19810, U.S.A.) at the ratio of OD A260/A280. RNA would be adopted for subsequent analyses only when it had a ratio close to 2.0.

Reverse transcription-polymerase chain reaction (RT-PCR) was performed to synthesize cDNA from the obtained RNA using Transcriptor First Strand cDNA Synthesis Kit (Roche, Vilvoord, Brussels, Belgium). This process was as per the manufacturer’s protocols. qRT-PCR was conducted to evaluate relative expression level of *HSDL2* mRNA using SYBR Green I Master Mix kit (Invitrogen, Carlsbad, CA, U.S.A.) on a 7300 Real-Time PCR System (Applied Biosystems, Foster City, CA, U.S.A.). *GAPDH* was used as the internal control gene. All of the primer sequences were as follows: *HSDL2* 5′-AAGCCACTCAAGCAATCTATCTG-3′ (forward) and 3′-GCTCTCCATATCCGACATTCCC-3′ (reverse); *GAPDH* 5′-TGACTTCAACAGCGACACCCA-3′ (forward) and 5′-CACCCTGTTGCTGTAGCCAAA-3′ (reverse). Final relative expression of *HSDL2* mRNA was calculated with the 2^−ΔΔ*C*^_t_ method and normalized to *GAPDH*. Three separate cell culture samples were subjected to analysis in triplicate.

### Cell viability assay

After the transfection, cell viability of the K1 and B-CPAP cell lines were detected with Celigo cell counting (Nexcelom Bioscience, Lawrence, MA, U.S.A.) and MTT assay. Logarithmic transfected cells were collected and digested using trypsin to obtain cell suspensions. The cell suspensions were seeded into a 96-well plate (2000 cells/well) and incubated in a humidified chamber with 5% CO_2_ at 37°C. The Celigo cell counting was performed once a day and lasted for 5 days. Cell number was recorded for the plotting of cell growth curve.

In MTT assay, cell suspensions were cultured in 96-well plates (1 × 10^4^ cells/well) and incubated in a humidified chamber with 5% CO_2_ at 37°C. Each well was added with 20 µl MTT (5 mg/ml, Sigma) at the time points of 1, 2, 3, 4 and 5 days and continually cultured at 37°C for 4 h. Then the culture medium was removed and 100 µl DMSO (Sigma–Aldrich, St. Louis, Missouri, U.S.A.) was added into every well. Absorbance at 490 nm was measured for the cell cultures. Day 1 meant 24 h after transfection. The values for day 1 were recorded as 1, and relative fold changes at each time point were recorded. All of the cell experiments were performed in triplicate using three separate cell cultures.

### Cell cycle and apoptosis assay

Cell cycle and apoptosis analyses of PTC cells were carried out using flow cytometry. K1 and B-CPAP cell lines after transfection were fixed with 75% ethanol for at least 1 h and washed using D-Hanks. Then the cells were stained with PI (Sigma–Aldrich, St. Louis, Missouri, U.S.A.) for 30 min. After the incubation at 4°C, the cells were detected using FACS Calibur flow cytometer (BD Biosciences, Franklin Lakes, NJ, U.S.A.). The obtained data were subsequently analyzed employing CellQuest software (BD Biosciences, Franklin Lakes, NJ, U.S.A.).

For the analysis on cell apoptosis, the apoptosis of the transfected cells were evaluated using Annexin V/propidium iodide detection kit (KeyGene, Nanjing, China) following the manufacturer’s instructions. FACS Calibur flow cytometer was used to detect the cells, and the data were analyzed using CellQuest software. Three separately prepared cell cultures were subjected to analysis in triplicate.

### Nude mice xenograft assay

The stud9y procedures were approved by the Experimental Animal Ethics Committee of Affiliated Hospital of the Academy of Military Medical Sciences.

In pre-experiment, the tumorigenicity of K1 and B-CPAP were tested. The PTC cells which were infected with Lentiviral vector GV115 were mixed with Matrigel at the ratio of 1:1. The cells of 1E+7 were injected into nude mice at armpit through subcutaneous injection. A total of six nude mice for the pre-experiments were randomly divided into two groups (three in each group), treated with K1 and B-CPAP cells, respectively. Tumor formation and growth capacity were examined within 8 days after injection. Twenty-eight days after injection, the mice were killed, and tumors were then isolated. Tumor weights and sizes were detected to estimate the tumorigenicity of the cells, and the tumors with a volume of 100 mm^3^ were considered to have stable tumorigenicity. Only cells with stable and high tumorigenicity were used for subsequent analyses.

#### Animal experiments

A total of 20 female BALB/c mice (4 weeks old, 18–20 g) were obtained from the Experimental Animal Center of Changchun Biological Institute (Changchun, China), and maintained under a specific pathogen-free condition with free access to water and food. The mice were randomly divided into two groups (ten mice in each group), namely *HSDL2* knockdown group (KD) and negative control group (NC). Matched cell suspensions containing sh*HSDL2* or shCtrl were given to nude mice at their armpits through subcutaneous injection. Each mouse received 1E+07 cells. Tumor growth was observed once a day after the injection, and tumor volume was recorded as well. Ten animals were used for analysis, and each sample was subjected to triplicate analyses.

Twenty-eight days later, the whole-body imaging was conducted for the mice. All of the mice were killed, and their tumors were isolated. The weights and sizes of the tumors were measured, and then they were fixed in paraformaldehyde.

### Potential target genes of *HSDL2* gene

In order to inspect molecular mechanism of *HSDL2* gene in PTC, potential target genes of *HSDL2* were predicted according to bioinformatics analyses using the GeneChip® PrimeView™ Human Gene Expression Array (Affymetrix, Santa Clara, CA, U.S.A.). Gene expression array assay was carried out by Shanghai Genechem Co., Ltd. (Shanghai, China). Three separately cultured cell samples were subjected to triplicate analyses. K1 cells were transfected by sh*HSDL2* and shCtrl vectors, respectively. The transfected cells were incubated in a humidified chamber with 5% CO_2_ at 37°C for 72 h. Then, the cells were harvested for gene expression array assay. In brief, total RNA sample was extracted from transfected K1 cells using TRIzol reagent (Invitrogen, Carlsbad, CA, U.S.A.) according to the manufacturer’s instructions. After purification, total RNA was used to prepare amplified RNA using GeneChip 3′IVT Express Kit (Affymetrix, Santa Clara, CA, U.S.A.). The amplified RNA was purified and fragmented for hybridization on gene expression array. Then raw data were analyzed using Partek® Genomics Suite (Partek Incorporated, St. Louis, U.S.A.), and *P*-values less than 0.05 were considered significant. Genes with a fold change more than 3 were chosen for pathway analysis using IPA software (www.ingenuity.com). IPA, an integrated online analysis software, can help understand gene expression data, microRNA data and small-scale experimental data. The software establishes a visualized experimental system that could construct interaction network between gene, protein as well as chemicals and drugs.

Then identified target genes were verified in PTC tissue specimens collected from all of the nude mouse models (ten mice in each group) using Western blotting. In KD group, proteins were extracted from PTC tissue specimens collected from ten mice, and mixed protein specimens were used for subsequent analysis. Same operations were performed for NC group. Each sample was subjected to triplicate analyses.

### Western blotting

Transfected cells were collected and lysed with 2× Lysis Buffer (Sigma–Aldrich, St. Louis, Missouri, U.S.A.). Total proteins in the cell or tissue samples were quantitated using BCA protein assay kit (Pierce Biotechnology, Rockford, IL, U.S.A.) following the instructions. The obtained proteins were first separated using 10% SDS/PAGE and then transferred on to polyvinylidene fluoride membranes (PVDF, Invitrogen, Carlsbad, CA). The membranes were blocked using TBST solution which was supplemented with 5% skim milk at 4°C for 1 h, and then incubated with first antibodies against *AKT3* (1:1000, CST#9272, Boston, U.S.A.), *NFATc2* (1:500, Abcam ab2722, Cambridge, U.K.), *PPP3CA* (1:2000, Abcam ab3673, Cambridge, U.K.), *FOS* (1:200, Abcam ab7963, Cambridge, U.K.) and *AKT1S1* (1:1000, CST#2691, Boston, U.S.A.) overnight at 4°C, followed by the incubation with second antibodies for 1.5 h at room temperature. The blots were visualized with enhanced chemiluminescence kit (GE Healthcare, Chalfont St. Giles, Buckinghamshire, U.K.) following the manufacturer’s protocols.

### Statistical analysis

All statistical analyses were performed using SPSS 18.0 software (SPSS Inc., Chicago, IL, U.S.A.). All experiments were carried out in triplicate. Quantitative variables were expressed as mean ± SD, and examined through one-way ANOVA test among multiple groups. Comparisons on quantitative data between the two groups were carried out using Student’s *t* test. Differences were considered statistically significant when *P*<0.05.

## Results

### Baseline characteristics of PTC patients

A total of 17 PTC patients including 6 males and 11 females were collected in our study. Their average age was 48.75 ± 8.62 years. Of them, 3 (17.65%) had tumor size more than 2 cm, and 10 (58.82%) had unilateral tumor. According to TNM staging, 13 (76.47%) patients were classified into I/II stages while 4 (23.53%) into III/IV stages. The basic characteristics of the patients are summarized in Table 1.

**Table 1:** The clinical characteristics of the PTC patients

Characteristics	Number of patients
Average age (years)	48.75 ± 8.62
Gender	
Male	6 (35.29)
Female	11 (64.71)
Tumor size, cm	
>2	3 (17.65)
≤2	14 (82.35)
Tumor location	
Unilateral	10 (58.82)
Bilateral	7 (41.18)
Multifocal tumor	
No	12 (70.59)
Yes	5 (29.41)
TNM stage (AJCC)	
I/II	13 (76.47)
III/IV	4 (23.53)

### Up-regulated *HSDL2* expression in PTC tissues and cell lines

**Figure 1:**
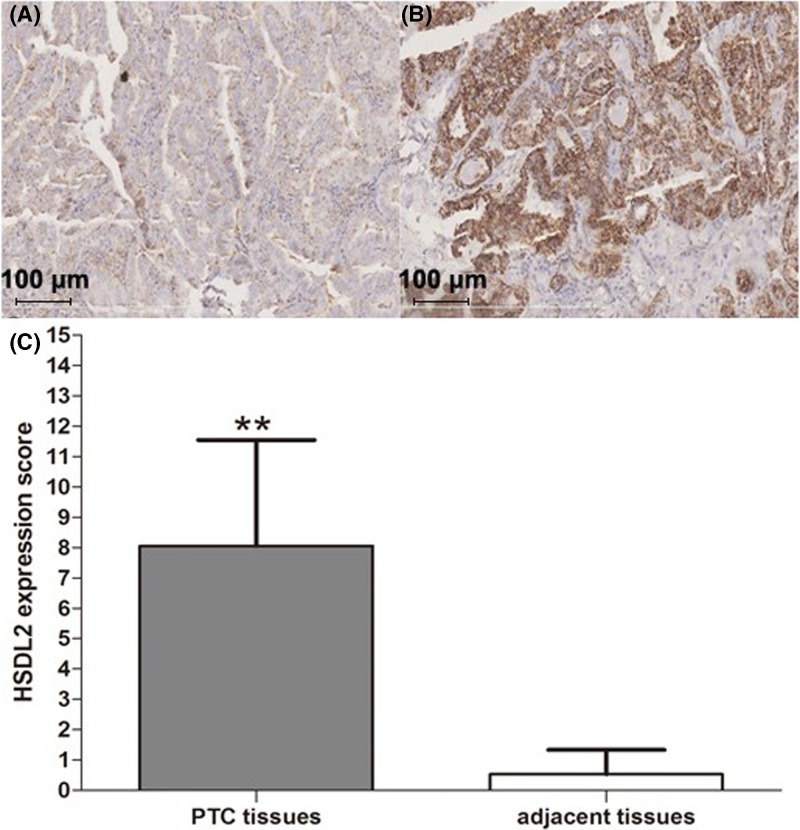
Expression of *HSDL2* protein was measured using IHC for tissue specimens collected from PTC patients (**A**) Representative IHC results on *HSDL2* expression for adjacent normal tissues. (**B**) The figure shows representative IHC assay result on *HSDL2* expression for PTC tissues. (**C**) Expression score of *HSDL2* was higher in PTC tissues than in adjacent normal ones. Three fields were randomly selected under microscope for each section. **: represented *P*<0.01.

Representative IHC results for *HSDL2* in adjacent normal tissues and PTC ones are shown in Figure 1A,B, respectively. Mean expression score of *HSDL2* protein was 0.50 ± 1.27 in adjacent normal tissues and 8.05 ± 4.71 in PTC ones, indicating that the expression level of *HSDL2* was significantly higher in PTC tissues than in adjacent normal ones (Figure 1C, *P*<0.01).

**Figure 2:**
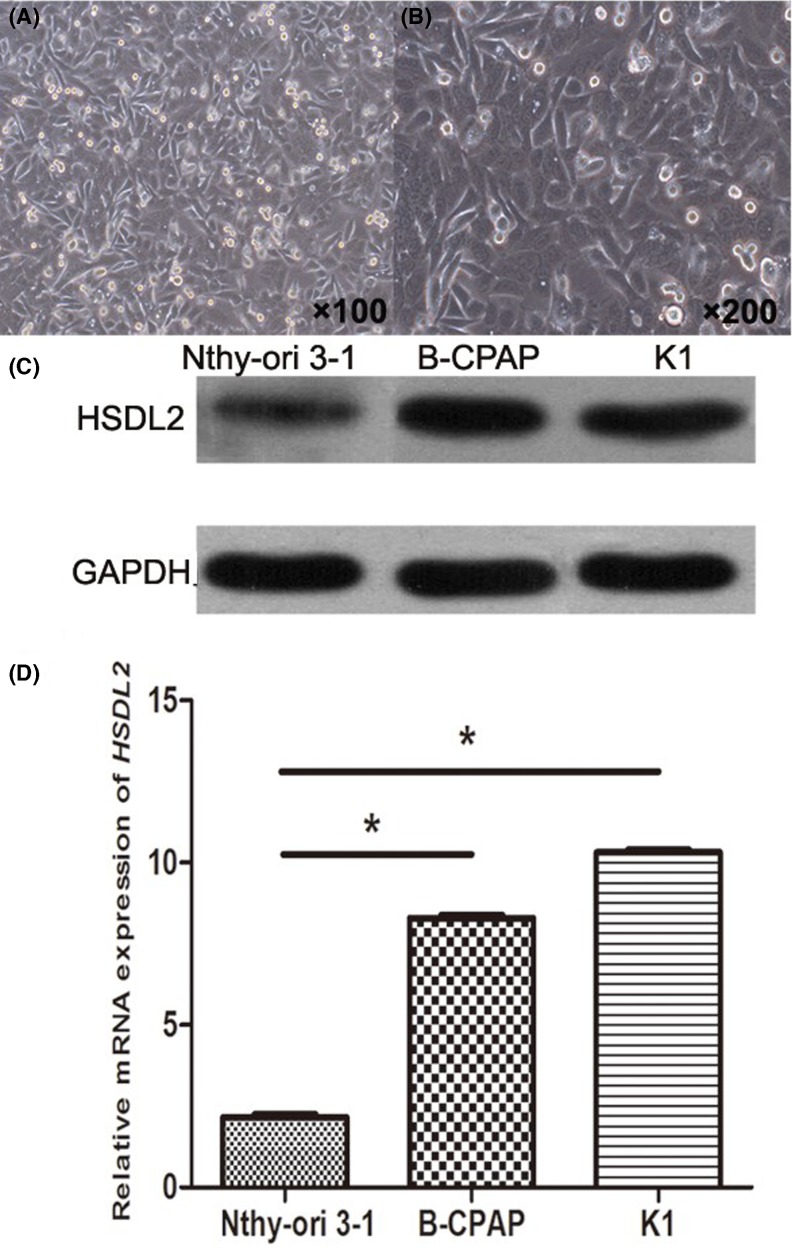
Expression pattern of *HSDL2* in PTC cell lines (**A,B**) Typical morphological observation on K1 cells under inverted microscope. The cells possess spindle or typical epidermal cell characteristics, and arrange closely. Only cells with typical morphologic parameters were used for subsequent analysis. (A) ×100; (B) ×200. (**C**) Representative figure for *HSDL2* protein expression in PTC cell lines. Compared with Nthy-ori 3-1 cell line, the levels of *HSDL2* protein obviously increased in PTC cell lines (K1 and B-CPAP cells). Three separately cultured cells were subjected to analysis in triplicate. (**D**) Relative expression of *HSDL2* mRNA was estimated through qRT-PCR for PTC cell lines (K1 and B-CPAP cells) and human normal thyroid cell line (Nthy-ori 3-1). The expression of *HSDL2* mRNA was significantly up-regulated in both the PTC cell lines in comparison with normal cell line (*P<0.05). Repeated analysis was performed with three separately cultured cell samples.

*HSDL2* mRNA expression and protein level in PTC cell lines (K1 and B-CPAP cells) and in human normal thyroid cell line (Nthy-ori 3-1) were also assessed. The cells were detected through cell morphology under microscope. Figure 2A,B show representative characteristics of K1 cell clone under inverted microscope. The cell clones possessed spindle or typical epidermal cell characteristics, and were arranged closely. Only cell clones with typical morphologic parameters were used for subsequent analysis. The level of *HSDL2* protein was obviously increased in PTC cell lines, compared with Nthy-ori 3-1 cell line (Figure 2C). Relative expression of *HSDL2* mRNA was remarkably higher in K1 and B-CPAP cells than in normal cells (all *P*<0.05, Figure 2D).

### Knockdown of *HSDL2* gene suppressed the proliferation of PTC cells

**Figure 3:**
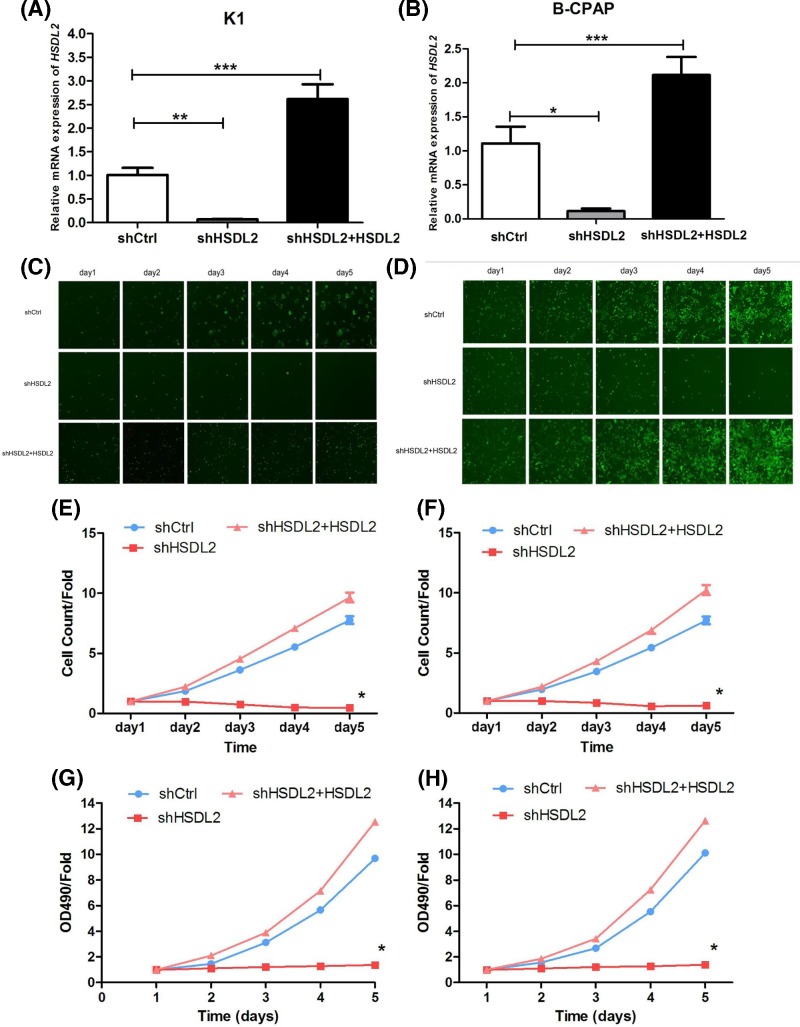
Knockdown of *HSDL2* gene suppressed the proliferation of PTC cells All repeated analyses were carried out using three separately prepared cell cultures. (**A**) *HSDL2* mRNA expression was down-regulated in K1 cells with sh*HSDL2* when compared with those transfected with shCtrl. (**B**) *HSDL2* mRNA expression was lower in B-CPAP cells transfected with sh*HSDL2* than those with shCtrl. (**C,D**) *HSDL2* knockdown inhibited cell growth in K1 ((C) ×200) and B-CPAP ((D) ×200) cells. (**E,F**) Quantitative results of Celigo cell counting. (**G,H**) Cell absorbance of K1 and B-CPAP measured via MTT analysis. The proliferations of K1 (E) and B-CPAP (F) cells were inhibited by down-regulated expression of *HSDL2*. The figures exhibit relative fold changes, and the OD490 value at day 1 (recorded as 1) was 0.1. Day 1 referred to 24 h after transfection. The results of rescue experience showed that the overexpression of *HSDL2* in sh*HSDL2* cells could reverse the role of *HSDL2* silencing in PTC cell progression.*P<0.05, **P<0.01 and ***P<0.001 represented the significant difference between the compared two groups.

In order to examine functional role of *HSDL2* gene in PTC, the gene was silenced using sh*HSDL2* in K1 cells and B-CPAP cells. After the transfection, relative expression levels of *HSDL2* mRNA were measured via qRT-PCR (Figure 3A,B). The results suggested that the levels of *HSDL2* in transfected cells were significantly decreased, revealing good transfection efficiency. Celigo cell counting and MTT assay were performed to explore the effect of *HSDL2* on cell proliferation. Celigo test results indicated that the numbers of both K1 and B-CPAP cells were decreased in sh*HSDL2* group when compared with shCtrl group (all *P*<0.05, Figure 3C–F). Besides, MTT results shown in Figure 3G,H indicated that the knockdown of *HSDL2* gene could significantly suppress cell proliferation both in K1 and B-CPAP cells, compared with control groups (all *P*<0.05). Meanwhile, we conducted the rescue experiences though the transfection of LV-*HSDL2* for overexpression in sh*HSDL2* transfected cells. The final results showed that overexpression of *HSDL2* in *HSDL2* silencing cells could significantly increased the proliferation of PTC cells (*P*<0.05) and reversed the effect of sh*HSDL2* on PTC cells.

### Knockdown of *HSDL2* gene induced cell cycle disorder and increased the apoptosis of PTC cells

**Figure 4:**
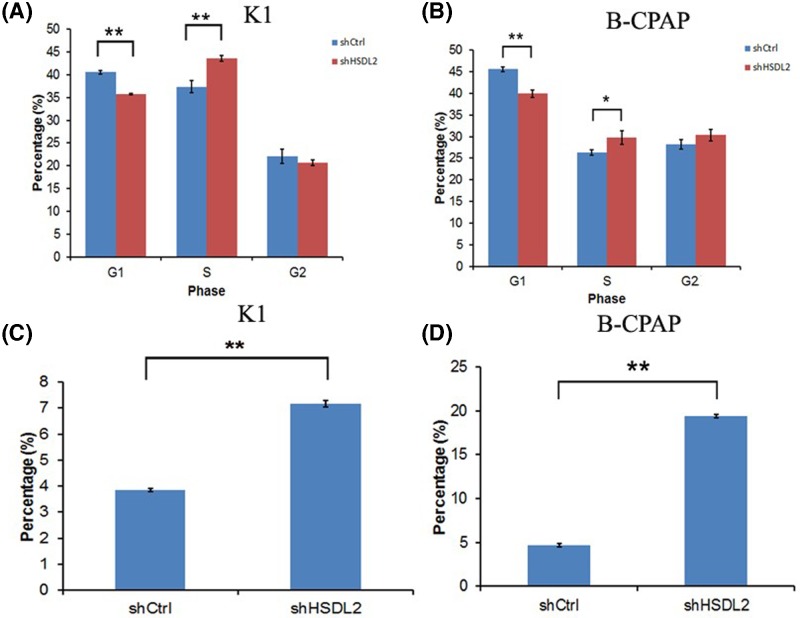
Effects of *HSDL2* gene on cell cycle and apoptosis of PTC cells (**A,B**) The percentage of G_1_ was decreased while the percentage of S was increased in K1 (A) and B-CPAP (B) cells transfected with sh*HSDL2*, compared with corresponding cells transfected with shCtrl (*P<0.05, **P<0.01). (**C,D**) Cell apoptosis was enhanced in both of *HSDL2*-knockdown K1 (C) and B-CPAP (D) cells in comparison with the controls (**P<0.01). All cell experiments were repeated in triplicate using three separate cell cultures.

Results from cell cycle analysis suggested that decreased *HSDL2* expression in both K1 and B-CPAP cells significantly reduced G_1_ period and increased S period (all *P*<0.05, Figure 4A,B). As shown in Figure 4C,D, the apoptosis percentages of K1 and B-CPAP cells were elevated in sh*HSDL2* group, compared with shCtrl group (all *P*<0.01) (Supplementary Figure S1).

### Reduced *HSDL2* expression inhibited PTC cell tumorigenicity *in vivo*

**Figure 5:**
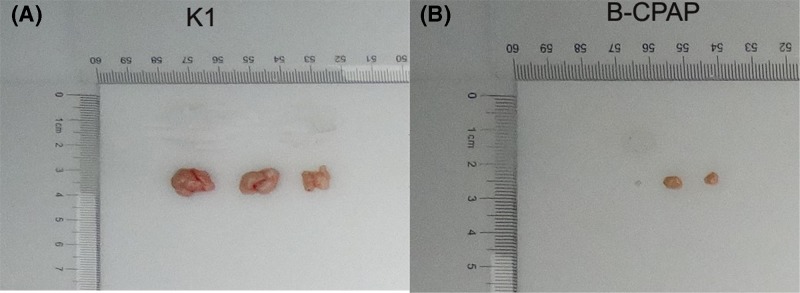
Tumor size detection in pre-experiments at day 28 after injection (**A**) Tumor size in PTC mice injected with K1 cells. (**B**) Tumor size in B-CPAP group. Twenty-eight days after injection, the size of B-CPAP cell induced-PTC was too small, with tumor volume less than 100 mm^3^, suggesting its weak tumorigenicity. Thus, B-CPAP cells were not suitable for subsequent analysis.

In our pre-experiment, the tumorigenicity of K1 and B-CPAP cells were tested. In K1 group, tumor formation was obvious in three nude mice 14 days after injection, and their tumor volumes were more than 100 mm^3^ 28 days after injection, suggesting tumorigenicity was moderate (Figure 5A). And K1 cells could be used for subsequent analysis. In B-CPAP group, tumor appeared in three mice after 15–20 days, suggesting weak tumorigenicity of B-CPAP cells. Moreover, the tumor volumes in these mice were less than 100 mm^3^ (Figure 5B). All if the data suggested that B-CPAP cells might be not suitable for animal experiments, and only K1 cells were chosen for subsequent analysis.

**Figure 6:**
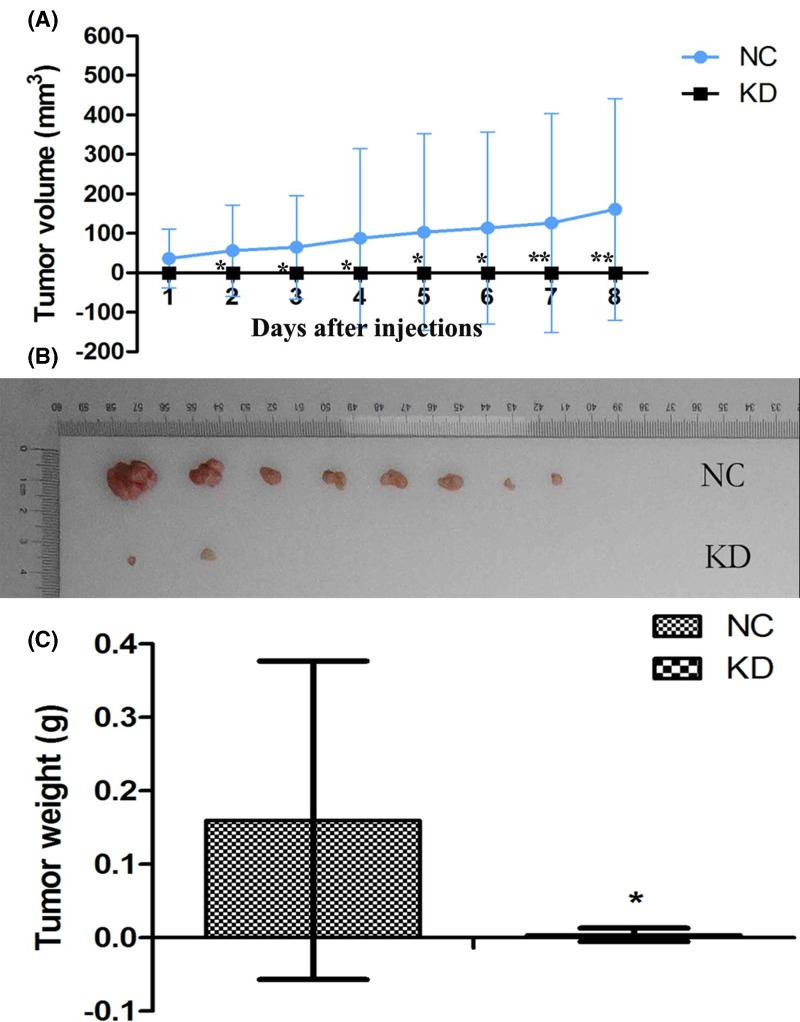
*HSDL2* knockdown inhibited PTC tumorigenicity *in vivo* (**A**) In PTC, down-regulated *HSDL2* expression could inhibit tumor volume (*P<0.05) in the first 8 days after cell injection. The data at each time point represented the average value of tumor volume for ten mice in each group. Each sample was subjected to triplicate analyses. At day 28 after cell injection, all of the nude mice were killed and their tumors were isolated to measure tumor sizes and weights. There were ten mice models in each group. (**B**) Tumor size was decreased in KD group (sh*HSDL2*) compared with NC (shCtrl) group. Due to relatively small tumor size, two samples in NC group and eight in KD group were invisible in the figure. (**C**) At day 28 after injection, tumor weight was significantly lower in KD group than in NC group (*P<0.05). Each sample was subjected to triplicate analyses.

K1 cells stably transfected with sh*HSDL2* (KD group) or shCtrl (NC group) were implanted into nude mice for tumor formation. As shown in Figure 6A, tumor volume was significantly less in KD group (injected with the K1 cells transfected with sh*HSDL2*) than in NC group in the first 8 days (*P*<0.05 for all). At day 28 after injection, tumor weights and sizes were estimated for nude mice in each group. As shown in Figure 6B,C, inhibiting the expression of *HSDL2* gene could obviously reduce tumor weights and sizes in animal models (*P*<0.05).

**Figure 7:**
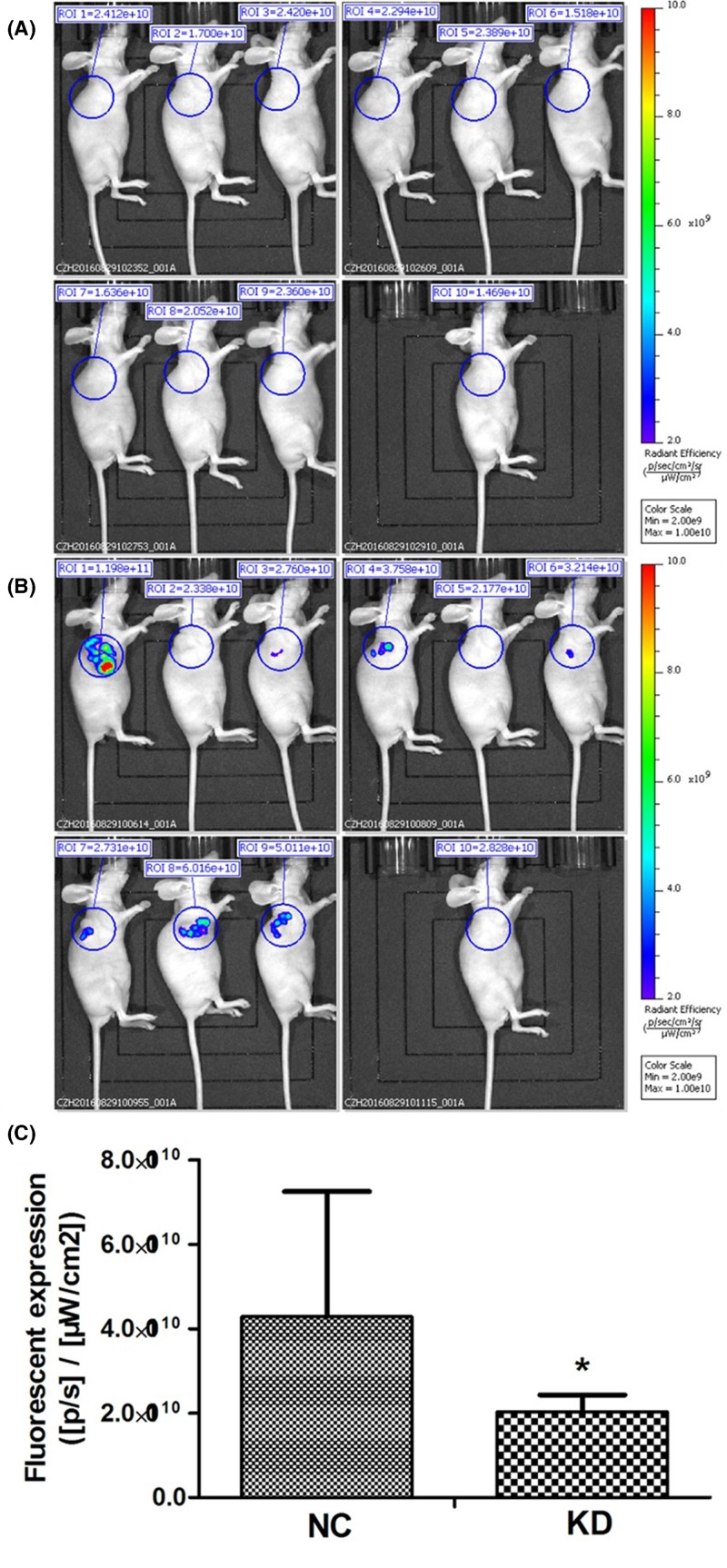
Whole-body imaging in nude mice xenograft assay (**A**) Whole-body imaging for the mice in KD group. One measure for each animal. (**B**) Whole-body imaging for the mice in NC group. One measure for each animal. (**C**) Fluorescent expression was remarkably lower in KD group than in NC group (*P<0.05).

To further confirm these results, whole-body imaging was obtained for the treated mice. As shown in Figure 7, fluorescent expression was remarkably lower in KD group than in NC group (*P*<0.05).

### *AKT3, NFATc2* and *PPP3CA* were potential targets of *HSDL2* in PTC

**Figure 8:**
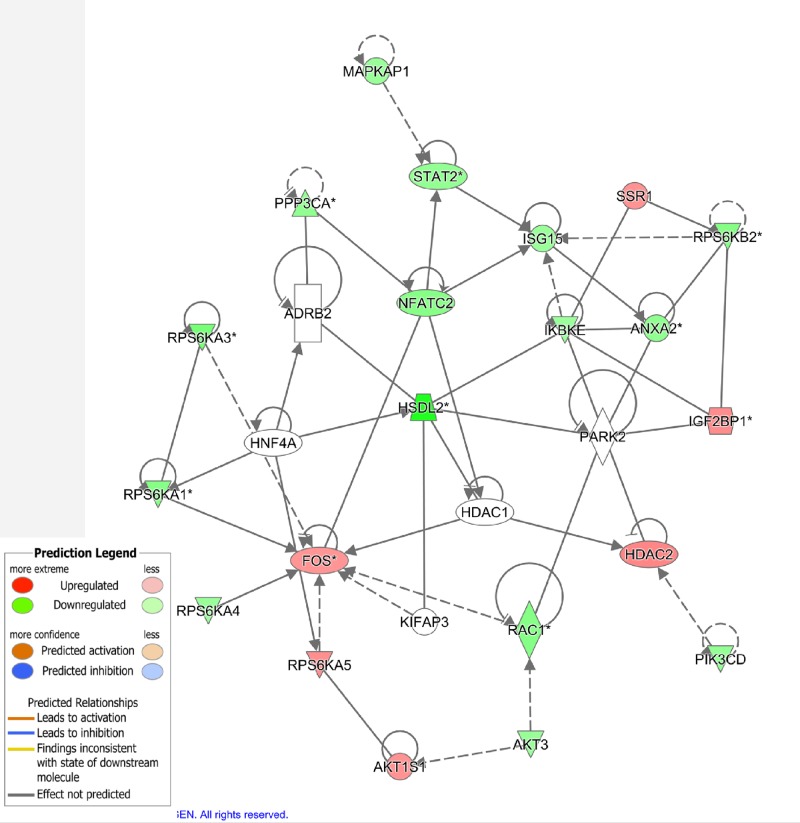
Gene interaction network obtained via bioinformatic analyses for *HSDL2* gene knockdown in K1 cell line Three separate cell cultures were used for analyses.

Human Gene Expression Array (Affymetrix, Santa Clara, CA, U.S.A.) presented a gene interaction network for molecular mechanism of *HSDL2* knockdown in K1 cell lines (Figure 8). From the figure we saw that the down-regulation of *HSDL2* gene could enhance the expression of *AKT1S1* and *FOS* while inhibit the expression of *AKT3, NFATc2* and *PPP3CA*.

**Figure 9:**
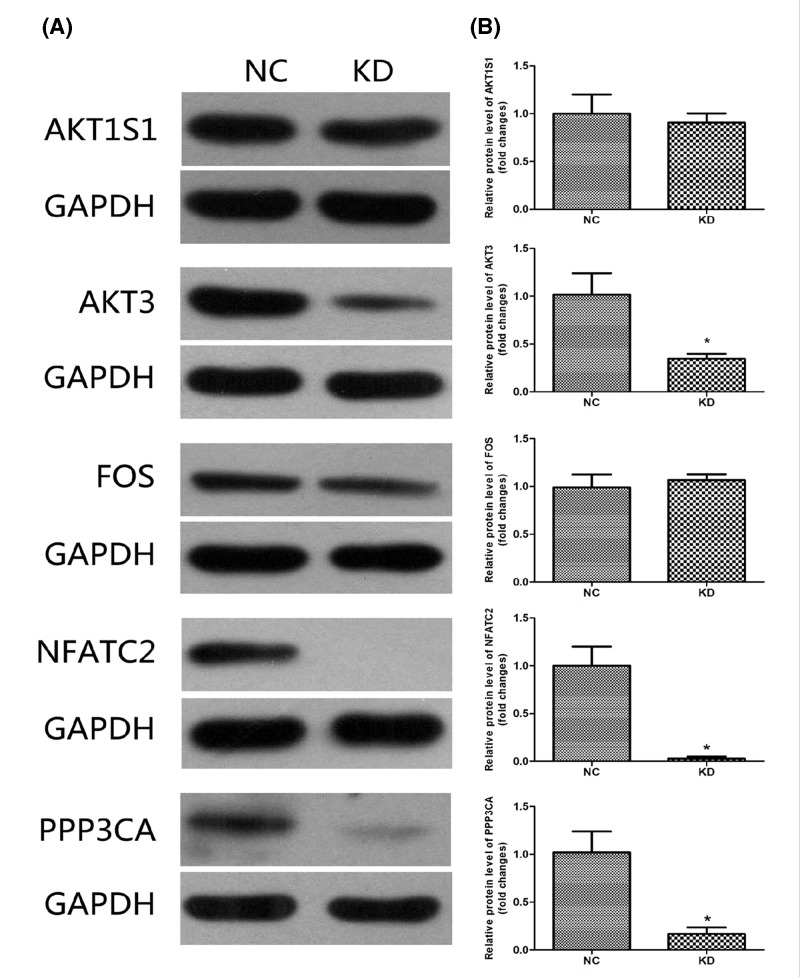
Expressions of downstream genes of *HSDL2* in mouse models A total of ten tissue samples were collected from each group (KD and NC groups). Each sample was analyzed three times. (**A**) Representative Western blotting results for five downstream genes. (**B**) No significant difference in the expressions of AKT1S1 and FOS proteins was found between KD and NC groups (all *P*>0.05). Protein expression of AKT3, NFATc2 and PPP3CA was remarkably down-regulated in KD group when compared with NC group (*P<0.05). Each sample was subjected to triplicate analyses.

Major downstream genes (*AKT1S1, AKT3, FOS, NFATc2* and *PPP3CA*) of *HSDL2* in the network were checked with Western blotting in tissue specimens collected from the nude mice (ten samples from each group). Analysis results indicated that *AKT3, NFATc2* and *PPP3CA* protein expressions were significantly lower in KD group than in NC group (all *P*<0.05, Figure 9).

## Discussion

As the most common type of thyroid cancers, PTC has received more and more attention because of its increasing morbidity and mortality worldwide. Benefited from considerable advancements in therapeutic technologies, PTC prognosis and outcomes witness significant improvements [21,22]. However, some PTC patients, approximately 10% of all the cases, are incurable due to metastasis and relative high tumor recurrence [23]. Therefore, it is critical to explore molecular mechanisms underlying PTC progression.

In previous studies, numerous cancer-related genes have been identified to be closely correlated with PTC progression [24–27]. For example, microRNA-126 was found to be down-regulated in PTC tissues and cell lines, while its up-regulation could suppress cell proliferation, migration and invasion, and promote the apoptosis of PTC cells [28]. Up-regulated expression of lysine-specific demethylase 1 (*LSD1*) has been detected in PTC cells, and its knockdown inhibited cell proliferation, tumorigenicity and invasion [29]. The growth and migration of PTC cells have been enhanced by the overexpression of hematopoietic pre-B cell leukemia transcription factor (PBX)-interacting protein (*HPIP*) [30]. In addition, Di Maro et al. [31] found positive relationship for elevated expression of anterior gradient protein 2 (*AGR2*) with increased migration and invasion capacity of PTC cells, which predicted poor overall survival of the patients. All these researches indicated the importance of genes in cancer development.

*HSDL2* belongs to SDR superfamily which exerts important influences on the metabolism of sugars, retinoids, steroids, xenobiotics and fatty acids [32]. Evidences have shown that *HSDL2* played a potential role in lipid metabolism [33,34]. Lipid metabolism is an important event in tumor development, and has been reported to be elevated in some cancer cells. Thus, it is reasonable to hypothesize that *HSDL2* might be involved in tumor progression. *HSDL2* up-regulation has been observed in human glioma samples, and reported to have close relationship with tumor proliferation, cell cycle and apoptosis [14]. However, the role of *HSDL2* in PTC remains poorly known until now. In the present study, we investigated the expression patterns of *HSDL2* gene and protein in both PTC tissues and cell lines, as well as its functional role in PTC development.

In our research, *HSDL2* protein level was higher in PTC tissues than in adjacent normal ones. Similarly, evaluated expressions of *HSDL2* mRNA and protein were also observed in two PTC cell lines, compared with normal human thyroid follicular epithelial cell line. All of these results suggested an oncogenic role of *HSDL2* gene in PTC. In order to testify such effect, we inhibited *HSDL2* expression in PTC cell lines via Lentiviral vector transfection. And the knockdown *HSDL2* expression significantly decreased cell proliferation, and induced cell apoptosis in both K1 and B-CPAP cell lines. Furthermore, mice injected with *HSDL2*-knockdown K1 cells had smaller tumor size, volume and weight than those receiving shCtrl. All of these data suggested that as an oncogene, *HSDL2* could promote PTC progression. Inhibiting *HSDL2* expression might suppress aggressive behaviors of PTC cells, as well as its malignant progression. So *HSDL2* might be a potential therapeutic target for PTC. Such conclusion was in line with the results obtained in the study on glioma by Ruokun et al. [14]. It is worthy to note that B-CPAP cells also presented high expression of *HSDL2*, but their tumorigenicity *in vivo* was much low. The observation might imply that oncogenic mechanisms of PTC may be not induced merely by a single gene. Tumor promoting pathway induced by *HSDL2* in B-CPAP cells might be inhibited. The functions of *HSDL2* in PTC might be distinct in different types of tumor cells. Therefore, it is urgent to explore molecular mechanisms of *HSDL2* in PTC.

Bioinformatics analyses suggested that various genes were involved in the interaction network of tumor-promoting role of *HSDL2* gene in PTC. The knockdown of *HSDL2* gene in PTC could induce the down-regulations of AKT3, NFATc2 and PPP3CA proteins. And their corresponding genes were confirmed as candidate targets for *HSDL2* gene in PTC. *HSDL2* could promote aggressive progression of PTC via mediating the expression of AKT3, NFATc2 and PPP3CA. AKT3, a member of AKT family, is involved in the regulations of various cellular processes, such as cell proliferation, migration, invasion and apoptosis. Its overexpression was observed in a variety of human cancers, including prostate cancer, glioma and PTC [35–37]. Reportedly, AKT3 up-regulation contributed to malignant progression in PTC [37]. Moreover, the study carried out in glioma demonstrated that *HSDL2* might regulate tumor progression through AKT-associated signaling pathway [14]. In the current study, we found that *HSDL2* knockdown weakened the expression of AKT3, suggesting that the oncogenic function of *HSDL2* in PTC might be correlated with AKT signaling pathways. NFATc2 is a calcium-responsive parlor, and potentially promotes cell invasion, migration, survival and angiogensis [38]. While PPP3CA protein is responsible for maintaining intracellular calcium homeostasis [39]. Alterations in these two proteins might contribute to malignant transformation of human cells, thus leading to tumors. Inhibiting *HSDL2* expression in PTC could decrease NFATc2 and PPP3CA, suggesting functional roles of *HSDL2* in PTC might implicate NFATc2 and PPP3CA proteins. However, it is worth noting that in gene expression microarray assay for K1 cells, the cell lines, showing similar characteristics as several other types of cancer cell lines, gradually lost some original properties. Thus, some PTC-specific genes targeted by *HSDL2* might be misdetected. The microarray analysis should be verified in original PTC cell line obtained from PTC tissues. In addition, *BRAF* (V600E) mutation, frequently observed in PTC, may influence the development and progression of PTC [40]. Besides, *BRAF* mutation may also affect gene expression in PTC cases [41,42]. In our study, K1 cells exert *BRAF* mutation, the tumor-promoting role of *HSDL2* gene, or its interaction network in PTC presenting BRAF-wt remained poorly known. The association between *HSDL2* and *BRAF* genes is rarely explored, but studies are still necessary to explore molecular mechanisms underlying the functional roles of HSDL2 in *BRAF* wild PTC.

The present study preliminarily investigated the functional roles of *HSDL2* gene in PTC, as well as the related mechanisms. Despite the encouraging results, there were still several limitations in the present study. First, the sample size was relatively small. Second, we found that the expression levels of *HSDL2* in PTC tissue and cell specimens were significantly increased, and that *HSDL2* knockdown could inhibit tumor growth. However, whether its overexpression in normal cells could induce tumorigenesis remained obscure. Moreover, Sun et al. [43] reported that *HSDL2* could also contribute to cell motility of ovarian cancer, revealing its promoting effects on tumor metastasis. Whether *HSDL2* was involved in early metastasis of PTC required further investigation. Third, although growing evidences have demonstrated that *HSDL2* gene could influence lipid metabolism, such effect was not detected in our study. Whether *HSDL2* regulated PTC progression via its function on lipid metabolism remained unclear. Fourth, microarray expression analysis was only performed in cell experiments, and verified in PTC tissues obtained from animal models in our study. Some potential target genes might be missed. Further microarray expression analyses in *in vivo* model are necessary to improve our findings. In addition, exact molecular mechanisms underlying regulatory effects of *HSDL2* on AKT3, NFATc2 and PPP3CA expressions remained unclear, and so did the question that whether *HSDL2* mediated aggressive progression of PTC via regulating their expressions. Further researches will be needed to explore related molecular mechanisms. Therefore, well-designed studies with larger sample size are required to verify our observations.

In conclusion, *HSDL2* expression was significantly up-regulated in PTC tissues and cells. Knockdown of *HSDL2* gene could suppress cell proliferation and promote cell apoptosis for PTC cells, thus inhibiting tumor growth *in vivo*. Functional roles of *HSDL2* gene in PTC might be associated with *AKT3, NFATc2* and *PPP3CA* genes*. HSDL2* promoted PTC progression which could be employed as a novel therapeutic target for the cancer. The present study may be the first investigation to explore the expression pattern and function of *HSDL2* in PTC.

## Supporting information

**Supplementary Figure S1:** 

## Abbreviations

AJCC: American Joint Committee on Cancer
ECACC: European Collection of Authenticated Cell Cultures
HSDL2: hydroxysteroid dehydrogenase like 2
IHC: immunohistochemistry analysis
IPA: Ingenuity Pathway Analysis
KD: knockdown
NC: negative control
NFATc2: nuclear factor of activated T cells c2
OD: optical density
PI: Propidium iodide
PPP3CA: protein phosphatase 3 catalytic subunit a
PTC: papillary thyroid carcinoma
qRT-PCR: quantitative real-time polymerase chain reaction
SCP2: Sterol carrier protein 2
SD: standard deviation
SDR: short-chain dehydrogenase/reductase
TBST: Tris-buffered saline containing Tween-20
TNM: Tumor Node Metastasis
