# Supplementary figures and images for: Down-regulated HSDL2 expression suppresses cell proliferation and promotes apoptosis in papillary thyroid carcinoma
Source: Biosci Rep. 2019 Jun 4;39(6):BSR20190425. doi: 10.1042/BSR20190425 (PMC6549096; doi:10.1042/BSR20190425)

**A**  
**K1**

shCtrl

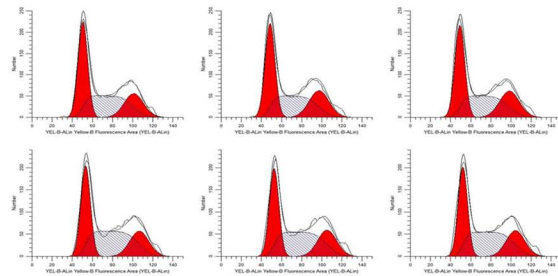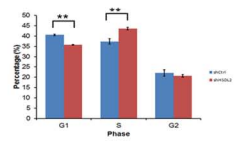

shHSDL2

**B**  
**B-CPAP**

shCtrl

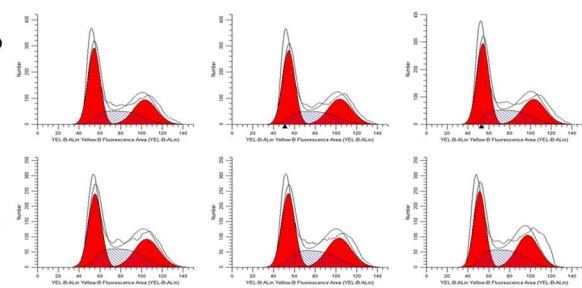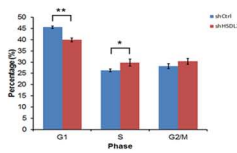

shHSDL2

**C**  
**K1**

shCtrl

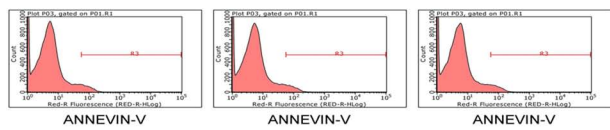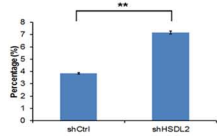

shHSDL2

**D**  
**B-CPAP**

shCtrl

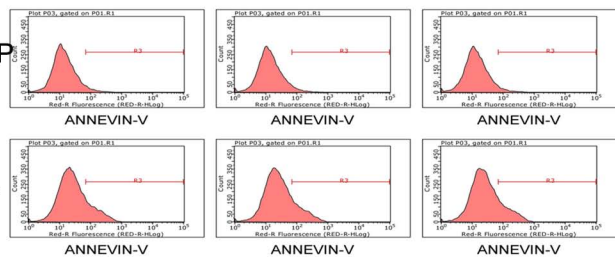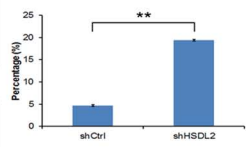

shHSDL2

Supplement: Supplementary file 1 [file bsr20190425_Supp1.pdf]
